# A mixed methods approach to understand variation in lung cancer practice and the role of guidelines

**DOI:** 10.1186/1748-5908-9-36

**Published:** 2014-03-22

**Authors:** Melissa C Brouwers, Julie Makarski, Kimberly Garcia, Saira Akram, Gail E Darling, Peter M Ellis, William K Evans, Mita Giacomini, Lorraine Martelli-Reid, Yee C Ung

**Affiliations:** 1 Department of Oncology, McMaster University, Juravinski Hospital Site, 1280 Main Street West, JHCC G207, L8S 4L8 Hamilton, ON, Canada; 2 Program in Evidence-based Care, Cancer Care Ontario, Hamilton, Canada; 3 Department of Clinical Epidemiology and Biostatistics, McMaster University, Hamilton, Canada; 4 University of Toronto, Toronto, Canada; 5 St. Joseph’s Health Care Centre, Hamilton, Canada; 6 Juravinski Hospital and Cancer Centre, Hamilton, Canada; 7 Centre for Health Economics & Policy Analysis, McMaster University, Hamilton, Canada; 8 Sunnybrook Odette Cancer Centre, Toronto, Canada

**Keywords:** Practice guidelines, Clinical decision making, Practice variation, Non-small cell lung cancer, Quality of care

## Abstract

**Introduction:**

Practice pattern data demonstrate regional variation and lower than expected rates of adherence to practice guideline (PG) recommendations for the treatment of stage II/IIIA resected and stage IIIA/IIIB unresected non-small cell lung cancer (NSCLC) patients in Ontario, Canada. This study sought to understand how clinical decisions are made for the treatment of these patients and the role of PGs.

**Methods:**

Surveys and key informant interviews were undertaken with clinicians and administrators.

**Results:**

Participants reported favorable ratings for PGs and the evidentiary bases underpinning them. The majority of participants agreed more patients should have received treatment and that regional variation is problematic. Participants estimated that up to 30% of patients are not good candidates for treatment and up to 20% of patients refuse treatment. The most common barrier to implementing PGs was the lack of organizational support by clinical administrative leadership. There was concern that the trial results underpinning the PG recommendations were not generalizable to the typical patients seen in clinic. The qualitative analysis yielded five themes related to physicians’ decision making: the unique patient, the unique physician, the family, the clinical team, and the clinical evidence. A dynamic interplay between these factors exists.

**Conclusion:**

Our study demonstrates the challenges inherent in (i) the complexity of clinical decision making; (ii) how quality of care problems are perceived and operationalized; and (iii) the clinical appropriateness and utility of PG recommendations. We argue that systematic and rigorous methodologies to help decision makers mitigate or negotiate these challenges are warranted.

## Introduction

Practice guidelines (PGs) are tools that contribute to changing care processes and the outcomes of care [1,2]. Clinical data audits and measures of practice patterns are methods often used to gauge the extent of adherence to recommendations and to identify ongoing quality issues and quality improvement successes (or failures). Studies looking at adherence to PG recommendations often show results that are interpreted as reflecting less than stellar performance. As a consequence, the full potential of PGs not being achieved is the conclusion [3,4].

In Ontario (population approximately 13.5 million), the cancer system is overseen by Cancer Care Ontario (CCO), an advisory body to the Ontario Ministry of Health and Long-Term Care on all matters related to cancer. CCO uses its performance improvement cycle as an organizing quality framework [5]. The cycle is defined by four components: data (to measure how the system is performing), knowledge (evidence-based knowledge on how the system should perform), transfer (the transfer of this knowledge to various stakeholders), and performance management (tools to facilitate application of knowledge). The Program in Evidence-based Care (PEBC) at CCO develops evidence-based PGs, and other evidence-resources, through a strategy of collaboration and application of rigorous methods [2,6]. Its contributions fit primarily in the knowledge and transfer quadrants of the cycle and directly inform the other quadrants, for example, recommendations serving as the foundation upon which quality indicators for the data quadrant are made.

It is through an analysis of this cycle that clinical quality concerns were identified related to two PG documents targeting the clinical management of the non-small cell lung cancer (NSCLC) patients. Specifically, chemotherapy for stage II/IIIA resected NSCLC patients (PG1) and chemoradiation for stage IIIA/B unresected NSCLC patients who also had good performance status and minimal weight loss (PG2) are recommended [7,8]. However, an analysis of practice patterns data in Ontario, Canada, as illustrated in the 2010 to 2011 Cancer System Quality Index (CSQI), a yearly report card of the quality of the Ontario cancer system, showed diverse patterns of practice in relation to these lung cancer PG recommendations [9]. Specifically, 41% of resected stage II/IIIA patients and 25% of unresected stage IIIA/B patients treated at regional cancer centres (RCC) received care that aligned with the PG recommendations. On average, 38% and 34% of resected stage II/IIIA Ontario patients and unresected stage IIIA/B Ontario patients, respectively, were reported to have received no treatment. Finally, practice patterns varied significantly across the different regions in the province for both of these patient populations (see Tables 1 and 2).

**Table 1:** Percentage of resected NSCLC stage II and IIIA patients treated with practice guideline recommended adjuvant chemotherapy following surgery by Local Health Integration Network (LHIN) Region of Residence (patients diagnosed in 2009)

**LHIN**	**Guideline treatment (RCC)**	**Treated non-RCC**	**Alternate treatment**	**No treatment <120 days of Surgery**	**Total resected II&IIIA cases**
1	32%	6%	3%	59%	34
2	38%	4%	4%	53%	68
3	16%	19%	19%	47%	43
4	46%	2%	7%	46%	61
5	10%	41%	10%	38%	29
6	32%	27%	5%	35%	37
7	29%	32%	5%	34%	65
8	40%	25%	3%	32%	68
9	49%	11%	6%	34%	83
10	52%	4%	11%	33%	27
11	61%	1%	7%	31%	72
12	37%	24%	11%	28%	46
13	50%	4%	4%	42%	50
14	65%	0%	10%	25%	20
Ontario	41%	14%	7%	38%	703

NSCLC, non-small cell lung cancer; RCC, Regional Cancer Centre.

Report date: December, 2011.

Data source: Cancer Care Ontario, ALR, OCR.

Notes:

Many patients in the “No Treatment” category may not be medically fit for the practice guideline treatment due to factors we are not currently able to adjust for. Patients may also decline treatment for personal reasons. Others may have been treated outside Ontario.

Alternate Treatment: cases receiving a therapy different from that recommended in the practice guidelines. This may include non-platinum-based chemotherapy or radiation therapy only.

Treated Non RCC: patients receiving chemotherapy outside of a cancer centre where the drug regimen is not reported to Cancer Care Ontario (CCO).

Parts of this material are based on data and information compiled and provided by the Canadian Institute for Health Information. However, the analyses, conclusions, opinions and statements expressed herein are those of the author, and not necessarily those of the Canadian Institute for Health Information.

**Table 2:** Percentage of unresected NSCLC stage IIIA and IIIB patients treated with practice guideline recommended chemo-radiation therapy by Local Health Integration Network (LHIN) Region of Residence (patients diagnosed in 2009)

**LHIN**	**Guideline treatment (RCC)**	**Treated non-RCC**	**Alternate treatment**	**No treatment < 180 days of diagnosis**	**Total nonresected IIIA & IIIB cases**
1	36%	6%	27%	31%	84
2	35%	6%	24%	34%	140
3	25%	13%	34%	28%	53
4	25%	1%	41%	33%	181
5	6%	6%	39%	48%	31
6	21%	11%	32%	36%	85
7	10%	16%	35%	38%	79
8	12%	15%	27%	46%	95
9	21%	12%	25%	41%	138
10	34%	0%	35%	31%	68
11	32%	2%	41%	25%	128
12	19%	11%	38%	32%	47
13	28%	6%	46%	20%	69
14	18%	3%	50%	29%	38
Ontario	25%	7%	34%	34%	1,236

NSCLC, non-small cell lung cancer; RCC, Regional Cancer Centre.

Report date: November, 2011.

Data source: Cancer Care Ontario, ALR, OCR.

Notes:

“No Treatment” does not necessarily indicate inappropriate care. Many patients may not be medically fit for the practice guideline treatment due to factors we are not currently able to measure. Some patients may also decline treatment for a variety of personal reasons. Others may have been treated outside Ontario.

Alternate Treatment: cases receiving a therapy different from that recommended in the practice guidelines. This may include non-platinum-based chemotherapy or chemotherapy only or radiation therapy only.

Treated Non-RCC: patients receiving chemotherapy outside of a cancer centre where the drug regimen is not reported to Cancer Care Ontario (CCO).

Parts of this material are based on data and information compiled and provided by the Canadian Institute for Health Information. However, the analyses, conclusions, opinions and statements expressed herein are those of the author, and not necessarily those of the Canadian Institute for Health Information.

In response to this data, we were asked for advice on how to fix the problem – particularly given the high proportion of patients receiving no treatment. Thus, we undertook a study with the objective to engage with Ontario clinicians providing care to lung cancer patients to better understand the process of clinical decision making. Specifically, we sought answers to the following questions:

1. How do clinicians interpret and perceive the practice pattern data? Is there agreement about what constitutes a potential clinical problem?

2. How do clinicians interpret and perceive the PGs, specifically the evidence and the recommendations, which are designed to guide practice?

3. What are the barriers and enablers clinicians experience in implementing the PG recommendations?

4. What factors are taken into consideration as part of the decision making process? Where does the evidence and recommendations, as per the PGs, fit in?

## Methods

### Study context and overview

This study took place in Ontario, Canada. As described above, CCO is the provincial advisor on all issues related to cancer. The province is divided into 14 different Local Health Integration Networks (LHIN) that are responsible for direct clinical management. Radiation therapy is provided in the regional cancer centres, systematic therapy is provided in the regional cancer centres or community, and cancer surgery is provided in academic settings and hospitals throughout the province. The links between surgeons and the regional cancer programs in the province varies.

Two PGs developed by the Lung Cancer Disease Site Guideline Development Group of CCO’s PEBC were targeted for inquiry and are the subjects of this study [5,6]. (The two PGs are described in Additional files 1 and 2, respectively.) In brief, the first PG recommends cisplatin-based chemotherapy for stage II/IIIA resected NSCLC patients (PG1), and the second PG recommends cisplatin-based chemotherapy with radiation for unresected stage IIIA/B NSCLC patients with good performance status and minimal weight loss (PG2). The evidence that underpins these recommendations shows that there is a survival benefit for patients treated with the recommended approach.

A mixed methods approach was used to address the research objectives. Ethics approval was received from McMaster University Faculty of Health Sciences/Hamilton Health Sciences Research Ethics Board (REB# 11–327). The project was funded by CCO, but the research team was editorially independent of the funders.

### Survey

#### Participants

Surgeons, medical oncologists, and radiation oncologists from Ontario were identified through multiple sources, including regional network leaders and network members in each of systemic therapy, radiation therapy, and surgery programs; individuals involved in lung cancer PG development and its application; and practicing clinicians who treat lung cancer. Participants were e-mailed a letter of invitation outlining the study objectives and participation requirements; letters were cosigned, where appropriate, by the provincial clinical leader at CCO.

### Process

Clinicians agreeing to participate completed the survey on an on-line password-protected platform. Participation was voluntary and confidential, and completion of the survey indicated consent to participate. Reminder e-mails were sent out on weeks two, four and eight after the initial invitation was distributed.

### Survey

The survey consisted of the following areas of inquiry:

1. Assessment of the PG Recommendations and the Evidentiary Base. Participants reviewed the abridged version of the PG and completed a survey relating to the recommendations (participants’ awareness and agreement with the PG recommendations, perceptions about their quality and implementability, and assessment of alignment with his/her clinical practice) and the evidentiary base underpinning the PG recommendations (completeness, validity). Each of these items was answered using a 7-point response scale (1 = totally disagree to 7 = totally agree).

2. Perceptions of Practice Patterns. For each PG, the overall provincial and regional practice pattern data from the CSQI were presented to the participant and summarized proportion of patients receiving no treatment, treatment adherent to PG recommendations, alternative treatment, and treatment outside of the cancer centre. The participant was asked to what extent the patterns aligned with his/her expectations and if they were clinically appropriate or indicative of a quality of care problem.

3. Benchmarks. Based on his/her own clinical experience, the participant was asked to estimate (a) the proportion of patients (in both populations) who refuse treatment even though they are appropriate candidates and (b) the proportion of resected stage II/IIIA patients who are not appropriate candidates (due to poor performance status, comorbidities) for cisplatin-based chemotherapy.

4. Barrier Analysis. Participants were presented with a series of barriers and enablers to the adoption of PG recommendations and asked to indicate which ones they perceived to be true. The questions were informed by the BARRIERs scale [10,11].

5. Demographics. Participant demographics regarding age, discipline, setting of practice, and location of practice were collected.

### Data analysis

Descriptive and frequency analyses were undertaken and all members of the team participated in the interpretation of the results.

### Key informant interviews

#### Overview

A grounded theory approach was used for the key informant interviews [12]. Two specific questions were used to direct the inquiry:

1. How do Ontario physicians construct treatment decisions regarding care for their resected stage II/IIIA NSCLC patients and their unresected stage IIIA/B NSCLC patients?

2. What is the role of the knowledge products, such as the PEBC PGs, in the decision making process?

### Participants and process

Following methodological standards for qualitative inquiry [13], purposive sampling was undertaken using clinician discipline, region and setting as the criteria for selection. Sampling continued until theoretical saturation was reached (i.e., new data generated no new insights into the findings). Letters of invitation were sent to a selected sample of candidate participants. In-person or telephone interviews were scheduled for those who agreed to participate. The semi-structured interview guide was pilot-tested and revised, with the final version used to guide the interview (see Additional file 3).

### Data analysis

As per grounded theory methods [12], data analysis and data collection (including participant interviews, field notes, and analytic memos) proceeded iteratively, with the preliminary findings informing directions for subsequent interviews. Interviews were transcribed and coded following conventional grounded theory procedures and theory-building stages [14].

## Results

### Survey

#### Participants

A total of 71 responses were received (response rate 30%). The majority of participants were men; surgeons; aged between 35 and 60 years; from the Hamilton Niagara Haldimand Brant region; and working in a cancer centre.

### Assessment of recommendations and evidentiary base

For PG1 (resected stage II/IIIA) and PG2 (unresected stage IIIA/B), the specific recommendations were rated very favorably by the participants (see Table 3). Indeed, the response modes for each item for both PGs tended to be at the extreme ends of the scale. Also, there were favorable assessments of the evidentiary bases underpinning both PGs (see Table 4), with a slight preference for the evidentiary base for PG1. The differences in quality ratings between PG1 and PG2 were greater for the assessments of the evidentiary bases than for the assessments of the recommendations.

**Table 3:** Practice guideline 1: resected NSCLC stage II and IIIA patients

**Item**	**Rating percent **^**A**^			**Mean**	**Sd**	**Mode**
	**Disagree**	**Neutral**	**Agree**			
**Assessment of the Recommendations**						
a. Aware of the recommendations	4.9	2.4	92.7	6.3	1.2	7.0
b. Agree with the recommendations	5.0	0	95.0	6.3	1.1	7.0
c. Recommendations are unambiguous	7.5	7.5	85.0	5.9	1.5	7.0
d. Recommendations are supported by the evidence	2.5	0	97.5	6.4	0.9	7.0
e. Recommendations are current	7.7	2.6	89.7	6.0	1.3	6.0
f. Recommendations are easy to apply in their clinical context	2.6	10.3	87.2	6.2	1.3	7.0
g. Recommendations are too rigid for the patients they are intended	68.4	10.5	21.1	2.9	1.8	2.0
h. Recommendations do not align with how they typically manage these patients	80.0	5.0	15.0	2.4	1.8	1.0^a^
i. Recommendations apply to the patients they target	7.9	0	92.1	6.0	1.3	6.0
j. Recommendations are biased	82.9	9.8	7.3	2.0	1.6	1.0
k. Support for the recommendations	5.0	2.5	92.5	6.3	1.1	7.0
l. Clinical practice of respondent aligns with the recommendations	5.0	5.0	90.0	6.2	1.1	7.0
**Assessment of the Evidentiary Base**						
a. It is complete	7.7	5.1	87.2	6.0	1.2	7.0
b. It is convincing	2.6	5.1	92.3	6.1	1.0	7.0
c. It is informative	7.5	0	92.5	6.1	1.2	7.0
d. It is relevant to typical patients	12.5	2.5	85.0	6.0	1.6	7.0
e. It is strong	5.3	2.6	92.1	5.9	1.2	6.0
f. It is current	10.5	2.6	86.8	5.8	1.5	6.0^a^

NSCLC, non-small cell lung cancer.

^A^Disagree (ratings 1 to 3 on 7-point scale); Neutral (rating 4 on 7-point scale); Agree (rating 5 to 7 on 7-point scale).

^a^Multiple mode exists. The smallest value is shown.

**Table 4:** Practice guideline 2: unresected NSCLC stage IIIA and IIIB Patients

**Item**	**Rating percentage**^**A**^			**Mean**	**SD**	**Mode**
	**Disagree**	**Neutral**	**Agree**			
**Assessment of the Recommendations**						
a. Aware of the recommendations	10.0	2.5	87.5	6.0	1.6	7.0
b. Agree with the recommendations	5.4	5.4	89.2	6.0	1.2	7.0
c. Recommendations are unambiguous	5.3	7.9	86.8	5.8	1.2	6.0
d. Recommendations are supported by the evidence	5.3	2.6	92.1	5.9	1.0	6.0
e. Recommendations are current	2.6	10.5	86.8	5.8	1.2	6.0
f. Recommendations are easy to apply in their clinical context	5.3	7.9	86.8	5.8	1.3	6.0
g. Recommendations are too rigid for the patients they are intended	76.3	5.3	18.4	2.8	1.7	2.0
h. Recommendations do not align with how they typically manage these patients	81.6	7.9	10.5	2.3	1.5	2.0
i. Recommendations apply to the patients they target	2.6	5.3	92.1	5.9	1.0	6.0
j. Recommendations are biased	89.7	10.3	0	1.8	0.9	2.0
k. Support for the recommendations	2.6	7.7	89.7	6.1	1.0	6.0^a^
l. Clinical practice of respondent aligns with the recommendations	2.6	7.7	89.7	6.1	1.0	6.0
**Assessment of the Evidentiary Base**						
a. It is complete	5.3	13.2	81.6	5.7	1.3	6.0
b. It is convincing	2.6	10.5	86.8	5.7	1.2	5.0^a^
c. It is informative	2.6	7.9	89.5	5.9	1.2	7.0
d. It is relevant to typical patients	2.6	2.6	94.7	5.9	1.1	7.0
e. It is strong	5.4	13.5	81.1	5.5	1.3	6.0
f. It is current	5.3	18.4	76.3	5.7	1.4	7.0

NSCLC, non-small cell lung cancer.

^a^ Multiple mode exists. The smallest value is shown.

^A^ Disagree (ratings 1 to 3 on 7-point scale); Neutral (rating 4 on 7-point scale); Agree (rating 5 to 7 on 7-point scale).

### Perceptions of practice patterns

As it relates to PG1 (for resected stage II/IIIA patients), 61% of the participants reported that the percentage of patients receiving treatment according to recommendations was less than what they expected, 3% reported it was more than what they expected, and 36% reported that it was as they expected. Further, while 40% of respondents reported the percentage of patients receiving no treatment was too low to be clinically appropriate, 3% reported it was too high to be clinically appropriate, and 46% reported it was about right and clinically appropriate. When asked if the variation in the regional patterns of practice across the province was an indication of a quality of care problem, 30% of respondents disagreed, 48% of respondents agreed, and 23% of respondents were neutral.

With respect to PG2 (for unresected stage IIIA/B patients), 54% of participants reported that the percentage of patients receiving PG recommended treatment was less than they expected, 5% reported it was more than they expected, and 41% reported it was as they expected. Further, only 27% of respondents reported the percentage of patients receiving no treatment was too low to be clinically appropriate, 22% thought it was too high to be clinically appropriate, and 51% thought it was about right and clinically appropriate. When asked if the variation in the regional patterns of practice across the province was an indication of a quality of care problem, 36% of respondents disagreed, 37% of respondents agreed, and 26% of respondents were neutral.

### Estimating benchmarks

The range in participants’ estimates of resected stage II/IIIA patients not eligible for treatment because of poor performance status or comorbidities was large, but over half estimated that between 15% and 30% of patients would not be appropriate. Participants’ estimates of the proportion of resected stage II/IIIA patients who refuse treatment although they are appropriate candidates was again large, but almost two-thirds estimated that between 15% and 30% of their patients meet these criteria. Finally, over 90% of participants’ estimated that between 5% and 20% of unresected patient stage IIIA/B patients refuse treatment even when they are appropriate candidates for this treatment.

### Barrier analysis

The most commonly reported barriers to implementing the PG recommendations were lack of organizational support by clinical administrative leadership (e.g., clinical head of the medical oncology program) (31.7% agreement); slow referral process (30.8% agreement); beliefs that patients seen in clinic do not reflect the patients included in the studies comprising the evidentiary base (25% agreement); and perceptions that it is not easy for patients in the region to gain access to the recommended treatment (23.1% agreement). In contrast, an unreliable referral process to the cancer centre/specialist (10% agreement); inadequate medical expertise in the region (9.8% agreement); belief that PG recommendations are not cost effective (7.5% agreement); lack of clinical skill of respondent (4.9% agreement); and perceptions that adverse effects of treatment were unacceptable (2.7% agreement) were the barriers that garnered the least support by participants. See Table 5 for full barrier analysis results.

**Table 5:** Barrier analysis

**Item**	**Rating**			**Mean**	**Sd**	**Mode**
	**Disagree**	**Neutral**	**Agree**			
a. Surgeons are reluctant to refer patients to a medical oncologist and/or radiation oncologist.	85.0	2.5	12.5	1.8	1.4	1.0
b. The referral process to a cancer centre or cancer specialist is complex.	76.2	11.9	11.9	2.5	1.6	1.0
c. The referral process to a cancer centre or cancer specialist is slow.	53.8	15.4	30.8	3.6	1.8	3.0
d. The referral process to a cancer centre or cancer specialist is unreliable.	82.5	7.5	10.0	2.3	1.5	1.0
e. Personal lack of clinical skill to implement the recommendations.	90.2	4.9	4.9	1.6	1.3	1.0
f. Organizational support from the clinical administrator leaders exists in the institution to support the implementation of the recommendations.	31.7	9.8	58.5	4.5	2.3	6.0^a^
g. There is adequate medical expertise in their region to implement the recommendations.	9.3	4.7	86.0	6.1	1.6	7.0
h. The patients in the studies comprising the evidentiary base do not reflect the typical patient they seen in the clinic.	60.0	15.0	25.0	3.1	1.8	2.0
i. It is easy for patients in their region to access the recommended treatment	23.1	5.1	71.8	5.1	1.6	5.0^a^
j. The implementation of the recommendations will result in unacceptable levels of adverse effects for the typical resected stage II and IIIA NSCLC patients seen in practice.	86.5	10.8	2.7	2.1	1.1	2.0
k. Optimizing the treatment of lung cancer patients is not as much of an organizational priority in their care setting as is the treatment of patients with other cancer diagnoses.	66.7	17.9	15.4	2.6	1.7	1.0
l. The implementation of the recommendations will yield the anticipated benefits as per the recommendations/guideline.	66.7	17.9	15.4	2.8	1.6	2.0
m. The recommendations are not cost effective.	77.5	15.0	7.5	2.4	1.3	1.0

^a^ Multiple mode exists. The smallest value is shown.

### Key informant interviews

In total, 21 participants (6 medical oncologists, 4 surgeons/surgical oncologists, 6 radiation oncologists, and 5 healthcare administrators) were interviewed; similar findings emerged from interviews with both clinicians and administrators. Five theoretical categories emerged concerning: (i) the unique patient, (ii) the family, (iii) the physician, (iv) the clinical team, and (v) the clinical evidence. We found a dynamic interplay between these factors in both clinical encounters and the construction of treatment decisions.

### The unique patient

The unique patient theme was comprised of three factors, including specific clinical circumstances; demographics; and social and personal contexts. Age was mentioned frequently as a defining feature that determined appropriateness of the treatment options, particularly for the unresected stage IIIA/IIIB patients, where trade-offs between benefit and risk are much smaller than for resected stage II/IIIA patients. Also important in this theme were the specific personal and social circumstances of the patients and their wishes with regard to the decisions made. For example, patients living alone or who had no access for transportation were considered less favorable candidates for chemotherapy because of the demands of the regimens, propensity of adverse effects, and risks for toxicities that may be difficult to manage alone or without easy access to a care provider.

### The unique physician

This theme was defined by physicians’ experiences, knowledge and judgments; physician recommendations; and the decision making style. Of particular note was the fact that personal clinical styles of the physician (e.g., those favouring more aggressive or less aggressive treatment approaches) and experiences from previous patients (e.g., degree of benefit or severity of adverse effects previously observed) were important factors that influenced how care options were presented and what were the preferred PG recommendations of the individual physician.

### The family

The family played a significant role in the decision making process. Presence or absence of family support and an accessible social network influenced the options offered to the patients and the intent of care (e.g., palliative intent or not). Family members emerged as key influentials in determining what treatment options would be encouraged or discouraged. Attitudes and perceptions of recommended treatments were influenced by family members who had gone through a similar treatment or serious illness. For example, a positive experience yielding an ‘I am a survivor’ narrative would influence how they communicated options to their ‘patient’ family member. Family members also served as a larger window into the patient’s life and helped the physicians understand the patient’s daily functioning and what the patient’s lifestyle was like.

### The clinical team

The treatment decision making process for NSCLC patients is not an isolated event between the physician and patient, but instead is a dynamic process that involves engagement of specialists and colleagues. Participants indicated that this can be more or less formal (informal chats or multidisciplinary cancer conferences), but it is a valued element in the decision making process. This is particularly true of the unresected stage IIIA and IIIB patients, whose care needs are much more complex and who present in a much more heterogeneous fashion.

### The clinical evidence

There was a tendency for each physician to apply his/her interpretation of the medical literature and to find and extrapolate new data to apply to his/her individual patient’s case. This could be done implicitly or explicitly. The predominant treatment styles or practices (e.g., aggressive versus conservative) at an institutional- or regional-level also had a strong influence in how evidence was scrutinized, with normative influences (e.g., ‘this is how we do it here’) playing a very influential role, regardless of whether there was alignment with the evidence.

The CCO PEBC PGs were seen as key foundational tools to the decision making process. However, while the CCO Lung PGs do identify the characteristics of the patient group most suitable for treatment, these important caveats are defined by the patient eligibility criteria in the clinical trials comprising the evidentiary base. Indeed, the participants expressed concern that PGs typically tackle issues and provide guidance only on those topics for which there exists primary studies to inform practice. In contrast, the diversity of the actual patient population and the contexts in which care is provided are much greater than the circumstances that the primary studies capture. Thus, for example, the PG recommendations related to care for unresected stage IIIA/IIIB NSCLC are quite limiting given that the patients who present in real life are much more variable with respect to functional status and comorbidities than the stage IIIA/ IIIB patients reflected in the evidence and PG recommendations. This lack of connection between the real life patient and the study patient can undermine the value, relevance and utility of the PG.

## Discussion

When approached to undertake this project, our team was presented with practice pattern data assumed to be reflective of a quality of care problem. Of particular concern was that a significant proportion of patients received no treatment, only a modest percentage of patients received treatment that aligned with the PG recommendations, and considerable regional variation existed within each of the clinical care options. We believe this project speaks to several issues relevant to the practice and scholarship of knowledge translation: (i) defining whether a quality of care problem exists or not is more complex than consideration of practice pattern data alone; (ii) limitations to an evidence base and the recommendations it informs in a PG compound this problem; and (iii) systematic and rigorous methodologies to help decision makers mitigate or negotiate these challenges are warranted. These issues will be addressed below.

Not surprisingly, and articulated by participants, there are limitations with clinical databases on which the practice patterns are based. These limitations related to the completeness, validity and reliability of the data [15,16]. The assessment of cancer PG adherence, particularly in the case of NSCLC, requires a complete and accurate capture of a patient’s stage, functional status, and weight loss; these factors directly influence the selection of optimal treatment and determining which patients are the best candidates for recommended therapy. Although this information is obtained during the clinical encounter, it may or may not be captured routinely in electronic databases. Similarly, data regarding the use and results of multidisciplinary case conferences (MCC) are routinely not captured. MCCs, the practice of bringing specific patient cases (often complex cases, as would be expected with lung cancer treatment) and care plans to a multidisciplinary team for feedback, is a key strategy to help determine the most optimal, safe, and preferred therapy tailored to the individual [17,18]. MCCs were formally introduced in Ontario at a system level in 2008. How they are specifically operationalized and function varies according to LHIN and specific disease sites.

Together, the limitations with patient-specific outcomes and MCC processes, make fair and valid interpretations of practice pattern data more problematic. For example, while 30% of patients categorized as ‘no treatment’ is large, the practice pattern data do not differentiate between appropriate reasons for no treatment (e.g., poor functional status, significant weight loss, patient refusal) from inappropriate reasons for no treatment (e.g., patients not being offered treatment, undue influence of patient families). From the survey data, participants were most likely to estimate that between 15% and 30% of Stage II/IIIA patients are not appropriate candidates for recommended treatment and that between 5% to 20% of patients refuse treatment. While these data are subject to recall bias, we have no reliable province-wide information to provide reliable estimates of ‘no treatment’ patients who were simply not medically fit for treatment or the extent to which a multidisciplinary team came to a consensus on that plan of action. Improvements in electronic medical record technology and application of other, albeit expensive and time-intensive, methodologies (e.g., chart review) may help address some of these problems.

Practice pattern data challenges are compounded by limitations to the PGs themselves. Similar to what has been found elsewhere [19], two thirds of our survey participants disagreed that the application of the PG recommendations would yield the anticipated benefits as seen in the primary studies, and our key informants reported that the patients included in the clinical trials on which the PGs were based do not reflect the typical patients seen in a cancer centre. For the clinicians, their patients are commonly more elderly, frail, and have comorbidities that make administration of the PG recommended treatment difficult. Thus, some degree of practice variation would and should be expected. These data raised a seemingly important paradox between perceptions of PG quality, the perceptions of the clinical acceptability or utility of PG recommendations, and their purpose.

As was found in the original external review of the targeted PGs as part of its development process [7,8], survey participants gave favorable ratings to both the evidence base (i.e., the systematic review) and the recommendations. Similarly, the key informants reported that the PEBC PGs are an important component of the decision making process. Thus, while data show that PGs may be methodologically sound, credible, and of high quality, the clinical relevance or clinical validity of the recommendations may not be optimal. Indeed, current standards for PG development, reporting or appraisal (e.g., AGREE II [20], Institute of Medicine Standards [21]) target the entire guideline process.

More recent efforts have begun to focus directly on the interplay between evidence, its context, the system, and the recommendations. For example, several international collaborative research efforts (e.g., AGREE-REX [http://www.agreetrust.org], DECIDE [http://www.decide-collaboration.eu]) are interested in advancing the science and practice of PGs by creating resources, tools and platforms targeted specifically to the recommendations (vs. the entire PG development process) and optimizing their implementability. Others have focused tactics to inform at the policy level [22]. Efforts to accelerate and better coordinate these efforts are warranted. More evaluation is required to determine tactics and strategies that are more and less effective. Further, exploring innovative statistical methods to facilitate systematic reinterpretation of the evidence from a lens of its probabilistic limitations would also be an asset. This may be of particular importance at the policy and system levels, where decision makers may be less familiar with evidence and data, their strengths and limitations, and where, in the absence of alternatives, PGs may be viewed as something more than a tool to guide clinical practice.

The qualitative analysis demonstrates that while PGs and data are important, they represent only one component of a complex decision making process. In a healthcare system, quality often focuses on adherence to evidence (consider the CCO quality improvement cycle [5]). This is not surprising given we likely have the most robust, albeit still very incomplete, methods to do this. However, less understood is how to fit the individual patient, the unique clinicians, the family, the clinical team, and the evidence – together – into a quality sphere or metric. In an environment in which patient-centred care is becoming an increasingly important and prioritized goal, understanding what this means and how to achieve this will be important [23].

In our analysis, we believe that the strongest signal of a quality of care problem is not the overall provincial pattern data (though, here too, we are concerned) but rather the significant variation between regions in the province. While there are important normative differences (e.g., regions that are more or less aggressive in their treatment approaches), patient population differences (e.g., socioeconomic class linked to cancer risk and health-seeking behavior), and familial system differences (e.g., how cancer is perceived, discussed) that may account for some of the variability, can it account for the magnitude reported? The barrier analysis found, as has been seen elsewhere [19], that referral processes to the cancer centres or oncology specialists were perceived as slow (30.8%) and not always easy (23.1%). However, again, the data are able to take this analysis so far. We are unable to systematically and reliably deconstruct these barriers to a regional level. For example, we do not have a systematic strategy to differentiate between patients who were or were not referred to a cancer specialist following surgery, and we cannot identify individual clinicians or clinician teams who may favour one treatment stream over another. Yet, each of these components is important to the interpretation of the data or the decision making process, and these issues are important in determining next steps. The strategies to tackle a regional variation problem may be very different from strategies to tackle a PG limitation problem; strategies for action need to be carefully tailored to the problem to optimize success. Further research to help differentiate the relative contributions of different challenges would be welcome.

We believe this study highlights important issues to the practice and scholarship of knowledge translation as a whole. First, from a practice perspective, the limitations of practice pattern data may compromise the ability to fairly prioritize and choose from the range of quality of care problems that clinicians and policy makers can tackle, particularly in an environment of limited resources and where healthcare equity is valued. This problem can be compounded in environments where quality and completeness of data varies across disease conditions, creating an ‘unfair’ playing field and pitting one health condition against another. Similarly, a consequence of questionable external validity of PG recommendations may be the inadvertent and inappropriate prominence given to some care options over others. Decision makers, clinicians and patients may expect more dramatic outcomes based on recommendations.

Data limitations and PG limitations also present challenges to advancing knowledge translation as a scientific field. The field wants to contribute to the generation of new knowledge that will ultimately yield a measureable positive impact on patients and populations. Within the context of PGs, this may mean, for example, the introduction of a new technology (e.g., a PG recommending chemotherapy), examining its impact on processes of care (e.g., administration of chemotherapy to appropriate and eligible patients), and outcomes (e.g., increased survival). If the outcomes do not align with hypotheses or expectations (i.e., practice patterns are not as anticipated or survival rates do not increase), it is difficult to attribute the causal meaning to the findings (e.g., problems with the PG, problems with measures, etc.). Moreover, and importantly, a PG is not simply a product that provides recommendations. It is a social and scientific process that builds a culture of stakeholders (historically clinicians and now more so patients and decision makers) that are receptive to, understand strengths and limitations of, and are able to use evidence as part of their decisions [2,6]. Thus, understanding what mechanism in a PG is the ‘change agent’ is difficult to ascertain.

We would argue that disclosing the limitations of practice pattern data and PGs are not sufficient. This is done, and this is done often fairly well. What are required are credible and reasonable methods to understand how to account and navigate the limitations and the appropriate modifications to make in response. While progress has been made, a more coordinated plan of action to avoid duplication and maximize the limited resources to advance this goal is warranted. The scientific community needs to go beyond describing components or themes of contextualization (although this is an important first step) to specific action steps to address them. While avoiding an environment in which ‘the good becomes the enemy of perfect’ is important, the research community must not be complacent. Even in the best circumstances, there is the risk that PGs can be so routinized that the individual core messages from each report are lost. In response, derivative knowledge tools that reflect the key PG messages (for clinicians and for administrators) may be useful – reminders, use of champions or key influentials, problem-based learning, and other knowledge ‘boosters’ – to juxtapose the PG recommendations against clinical data to promote discussion, debate and change [24].

While one of the strengths of any PG is that it is linked to the evidence, paradoxically, this can also present a challenge, as discussed. The evidence from carefully designed phase III randomized controlled trials cannot account for the range of important comorbid conditions that may be commonly present at a clinic or the range of other factors such as the individuality of a given patient, the impact of family, and social or institutional contexts that may significantly influence what care option is offered or advised. Recognition of these factors, methods to facilitate a more transparent use of expert knowledge and consensus of opinion where this knowledge may best be found, and the greater use of pragmatic trials by primary researchers and subsequently by PG developers [25], may lead to a more fulsome discourse and more implementable recommendations. Benchmarks of adherence that adopt estimates and ranges of what constitutes reasonable variation would be advantageous to better contextualize clinical practice pattern data and to more accurately ascertain the risk that a quality problem exists.

A strength of our study is that we designed and applied high quality qualitative and quantitative methods to understand the problem. While the clinical context of this study targeted cancer and the jurisdictional context targeted Ontario, Canada, we believe that the issues raised are generalizable across disease types and jurisdictions. Many health authorities and governing health bodies (e.g., Unites States, the United Kingdom) rely on practice pattern data to determine healthcare priorities, monitor progress toward quality improvement goals, and direct investments and resources. All PG development groups confront limitations of the evidence base and challenges with generalizing evidence to the range of patients faced in the healthcare system.

A significant limitation of our study is that although appropriate methods were used to optimize response rates (co-signatures, repeated reminders, etc.), a disappointing response rate emerged with the quantitative component (30%), leading to the risk of a biased participant sample. However, for most of the surveys, the confidence intervals were small, and for most items, participants focused on the extremes of the scale. While this explanation should not mitigate all concerns about selection bias and low response rates, perhaps, as these data show, the absolute variability in perceptions is limited as well. A second limitation is the threat of recall bias, particularly in participants’ judgments about the estimated proportion of patients for whom treatment is not appropriate or who refuse treatment.

## Conclusion

This study used a mixed methods approach to better understand how clinical problems are defined and the role of PGs in addressing clinical problems and informing clinical decisions. Our study demonstrates the challenges inherent in (i) the complexity of clinical decision making, (ii) how quality of care problems are perceived and operationalized, and (iii) the clinical appropriateness and utility of PG recommendations. The PG community (i.e., developers, users, researchers) needs to accelerate the development of methods and strategies to make practice pattern data interpretation, PGs, and PG recommendations more clinically valid, relevant and useful.

## Competing interests

Dr. Brouwers is the Scientific Director of the Program in Evidence-based Care. Dr. Ellis and Dr. Yee are the co-chairs of the Cancer Care Ontario Provincial Lung Guideline Group of the Program in Evidence-based Care. No other competing interests were declared.

## Authors’ contributions

MB and JM conceived and designed the project. MB, JM and KG contributed to the development of the protocol of the quantitative study component. MB, SA, PE and MG contributed to the development of the protocol of the qualitative study component. MB, JM, KG and SA contributed to the acquisition of data. All authors contributed to the interpretation of the data, were involved in drafting the manuscript or revised it critically for important intellectual content, and have given final approval for the final draft. The qualitative study served as the thesis for SA as partial fulfillment for a Master’s of Science degree in McMaster University’s Health Research Methodology Graduate Program. MB served as her thesis supervisor.

## Supplementary Material

Additional file 1 Practice Guideline 1: Resected NSCLC Stage II and Stage IIIA Patients. Click here for file

Additional file 2 Practice Guideline 2: Unresected NSCLC Stage IIIA and IIIB Patients. Click here for file

Additional file 3 Interview Question Guide. Click here for file
