# Supplementary material for: A mixed methods approach to understand variation in lung cancer practice and the role of guidelines
Source: Implement Sci. 2014 Mar 22;9:36. doi: 10.1186/1748-5908-9-36 (PMC3998045; doi:10.1186/1748-5908-9-36)
Supplement: Additional file 1 — Practice Guideline 1: Resected NSCLC Stage II and Stage IIIA Patients. [file 1748-5908-9-36-S1.docx]

**Additional file 1: Practice Guideline A: Resected NSCLC Patients**

**Postoperative Adjuvant Chemotherapy, with or without Radiotherapy, in Completely Resected Non-Small Cell Lung Cancer (7-1-2)**

**Question**

Does the use of postoperative chemotherapy, with or without radiotherapy, in patients with completely resected non-small cell lung cancer improve survival? Toxicity was also an outcome of interest.

**Target Population**

These recommendations apply to adult patients with completely resected non-small cell lung cancer. A complete resection is defined as an R0 resection.

**Disease Stage-Specific Recommendations**

***Completely resected stage IA non-small cell lung cancer***

- In the opinion of the Lung Disease Site Group (DSG), adjuvant chemotherapy should not routinely be used in this patient population due to their good overall survival and because the evidence for a survival benefit with adjuvant chemotherapy is uncertain.
- Postoperative radiotherapy in combination with chemotherapy should not be used.

***Completely resected stage IB non-small cell lung cancer***

- Postoperative adjuvant platinum-based chemotherapy is not recommended for routine use in this population.
- Postoperative radiotherapy in combination with chemotherapy should not be used.

***Completely resected stage II non-small cell lung cancer***

- Postoperative adjuvant cisplatin-based chemotherapy is recommended in this population.
- Postoperative radiotherapy in combination with chemotherapy should not be used.

***Completely resected stage IIIA non-small cell lung cancer***

- Postoperative adjuvant cisplatin-based chemotherapy is recommended in this population.
- The role of postoperative radiotherapy is unclear in this stage of disease.

**Treatment Dose and Schedule**

- The National Cancer Institute of Canada Clinical Trials Group (NCIC-CTG) trial achieved a statistically and clinically significant survival benefit for adjuvant chemotherapy without postoperative radiotherapy using vinorelbine (25 mg/m2 weekly for 16 weeks) combined with cisplatin (50 mg/m2 given on days 1 and 8) for patients with stage IB or II (1,2). Those studies that have demonstrated benefit for adjuvant chemotherapy in stage IIIA disease used similar regimens. It is unknown whether other doses and schedules of administration of these agents will produce similar benefits. One of the drawbacks to a recommendation favouring the cisplatin-vinorelbine regimen used in both the NCIC-CTG and Adjuvant Navelbine International Trialist Association (ANITA) trials is that it involves a weekly administration of vinorelbine over 16 to 20 weeks. This is difficult for patients and providers alike. It is also not known whether more convenient treatment schedules such as the three-weekly administration of vinorelbine on days 1 and 8 and cisplatin on day 1 would have similar efficacy. The Lung DSG recommends that practitioners use the regimen and schedule that has produced the best current results in a randomized trial. If this is not possible to do, the Lung DSG recommends that medical oncologists select one cisplatin-based chemotherapy regimen to use consistently for all adjuvant lung cancer therapy, as this should optimize patient safety.

**Other Recommendations**

- The use of adjuvant chemotherapy involving alkylating agents is not recommended as it has been found to be detrimental to survival.
- In the opinion of the Lung DSG, a recommendation for or against the use of the adjuvant uracil-tegafur combination (UFT) in a North American population is not appropriate at this time because the drug combination has only been tested in lung cancer patients in Japan and the results may not be generalizable to non-Japanese populations. UFT is currently not available in North America.

**Qualifying Statements**

- Although the evidence for or against the use of postoperative radiotherapy in combination with chemotherapy is unclear, in the opinion of the Lung Cancer Disease Site Group, the combination treatment should not be used in stage I or II disease. This opinion is based on the lack of a clear survival benefit for chemoradiotherapy in comparison to radiotherapy alone, a strong survival benefit associated with chemotherapy alone in stage II disease (with uncertain evidence in the case of stage IB disease), and a survival detriment associated with radiotherapy alone. In the three trials of adjuvant chemoradiotherapy reviewed in this guideline, deaths associated with the combination treatment occurred in 2% to 9% of patients, while in the trials of adjuvant chemotherapy, chemotherapy-related deaths occurred in 0.8-2% of patients. However, the appropriateness of postoperative radiotherapy is less clear for stage IIIA disease: the two trials that included stage IIIA patients, and showed a statistically significant overall survival benefit for adjuvant chemotherapy, had methodological limitations in that they administered sequential radiotherapy according to centre choice.
- Insufficient evidence exists to identify specific subgroups of patients that may differentially benefit from the use of postoperative adjuvant platinum-based chemotherapy. Most adjuvant platinum-based chemotherapy trials have mainly involved patients with a good performance status (0-1) and included patients with a mix of disease stages (I-III); the only trial without postoperative radiotherapy that yielded an overall survival advantage only included patients with stage IB and stage II disease. In the two trials that administered postoperative radiotherapy according to centre choice and showed a statistically significant overall survival benefit for adjuvant chemotherapy, the survival benefit appeared to be greatest for stage IIIA patients from a forest plot of hazard ratios by disease stage and a comparison of survival curves by disease stage. However, no statistical analyses were reported by disease stage in either trial.
- The potential benefits, limitations, and toxicity of treatment should be fully discussed with the patient. Severe toxicities (grade 3 or 4) frequently associated with platinum-based chemotherapy include hematologic events, particularly neutropenia, nausea and vomiting, and fatigue. The Lung DSG believes that for stage II and IIIA disease, and for patients fit enough to receive chemotherapy, the survival benefits of adjuvant chemotherapy strongly outweigh the treatment toxicity.

**Key Evidence**

- Three large individual patient data meta-analyses and five analyses that pooled only published data, have detected a survival benefit in favour of some types of postoperative adjuvant chemotherapy.
- In the Non-Small Cell Lung Cancer Collaborative Group (NSCLCCG) individual patient meta-analysis, an absolute survival benefit of 5% at five years was detected for adjuvant cisplatin-based chemotherapy in patients with potentially curative resections of early-stage disease (eight trials, 1,394 patients). Although not statistically significant (hazard ratio [HR], 0.87; 95% confidence interval [CI], 0.74-1.02; p=0.08), that benefit would be considered clinically significant and has been confirmed in subsequent meta-analyses. In the 1995 meta-analysis, adjuvant chemotherapy involving alkylating agents in five trials (2,145 patients) was found to be detrimental to survival, with a 5% absolute reduction in survival at five years (HR, 1.15; 95% CI, 1.04-1.27; p=0.005). No survival benefit or detriment was detected for postoperative adjuvant chemotherapy in combination with radiotherapy when compared with surgery plus postoperative radiotherapy alone (HR, 0.98; p=0.76).
- In the Lung Adjuvant Cisplatin Evaluation (LACE) individual patient meta-analysis (five trials, 4,584 patients) an absolute survival advantage of 5.3% at five years was detected for cisplatin-based chemotherapy (HR, 0.89; 95% CI, 0.82-0.96; p=0.004). Subgroup analysis found the benefit varied by disease stage (stage IA: HR, 1.41; 95% CI, 0.96-2.09; stage IB: HR, 0.92; 95% CI, 0.78-1.10; stage II: HR, 0.83; 95% CI, 0.73-0.95; and stage III: HR, 0.83; 95% CI, 0.73-0.95).
- In the Hamada individual patient meta-analysis (six trials, 2,003 patients with completely resected disease), a statistically significant survival benefit was associated with surgery followed by oral UFT compared with surgery alone (HR, 0.74; 95% CI, 0.61-0.88; p=0.011), corresponding to an absolute increase in survival of 4.6% at five years.
- Among the 16 randomized controlled trials of postoperative adjuvant platinum-based chemotherapy, six involving more than 100 patients per treatment arm were published after the 1995 meta-analysis.
- The largest trial (n=1,867), compared cisplatin in combination with one of etoposide, vinorelbine, vinblastine, or vindesine, to a control arm of no chemotherapy, and detected a survival advantage for chemotherapy (HR, 0.86; 95% CI, 0.76-0.98; p<0.03) for patients with stage IB, II or III disease. Radiotherapy was administered according to centre choice and the survival advantage observed was not differentially associated with the use of radiation or disease stage (3).
- Two recent trials detected a statistically and clinically significant survival benefit for adjuvant cisplatin with vinorelbine compared with surgery alone. One trial administered cisplatin-vinorelbine to patients with stage IB or II disease, and found a 15% absolute benefit at five years (2). The second trial also administered cisplatin-vinorelbine and found an 8.6% absolute benefit at five years. That trial included patients with stage IB and II, as well as IIIA, and used radiotherapy according to centre choice. Radiation use was associated with increased mortality in univariate analysis (HR, 1.34; 95% CI 1.10-1.63, p = 0.003) (4).
- One trial published in abstract form administered carboplatin-paclitaxel to 344 patients with stage IB disease and did not detect a significant difference in overall survival (HR, 0.80; 90% CI, 0.60-1.07, p=0.10), although a significant difference was found in disease-free survival (HR, 0.74; 90% CI, 0.57-0.96, p=0.027) (5,6).
- The other two large trials, did not detect a statistically significant survival difference between treatments. Differences in trial characteristics (e.g., chemotherapy type, stage of disease, and use of radiotherapy) may have contributed to those conflicting results.
- There is strong evidence that UFT as a postoperative adjuvant chemotherapy improves survival in patients with stage I non-small cell lung cancer, particularly adenocarcinomas. However, nine of the 13 trials of adjuvant oral chemotherapy used UFT alone or in combination with other intravenous chemotherapy agents and included between 30 and 979 patients. All nine trials were conducted in Japan and involved primarily stage I disease (68-100% of patients). Among the five trials that compared adjuvant UFT-based combination chemotherapy with surgery alone, only one small trial detected a statistically significant survival benefit for adjuvant therapy (cisplatin-vindesine-UFT, p=0.045). In addition, two trials (>100 patients per treatment arm) detected a survival benefit for adjuvant therapy only after pretreatment prognostic factors were taken into account (cisplatin-doxorubicin-UFT, p=0.044; cisplatin-vindesine-UFT, p=0.037). Among the seven trials that compared adjuvant UFT, given postoperatively for one to two years, with no UFT, four detected a statistically significant survival advantage with UFT. The largest of those four trials, involving 979 patients with stage I adenocarcinoma, detected an absolute survival benefit of 3% at five years (HR, 0.71; 95% CI, 0.52-0.98; p=0.04).
- In the one study using cisplatin-vinorelbine without postoperative radiotherapy (1) patients experienced the following severe (grade 3 or 4) hematologic toxicities: neutropenia (73%), febrile neutropenia (7%), and anemia (7%).
- Common and severe non-hematologic toxicity associated with cisplatin-vinorelbine without postoperative radiotherapy included malaise or fatigue, 15%; nausea and vomiting, 7-10%; and anorexia, 10%. Treatment-related mortality associated with a cisplatin-vinorelbine combination regimen has been reported as 0.8% (7) and 2% (5). In a quality-of-life analysis, only adverse effects associated with neurotoxicity persisted after treatment was completed.

**REFERENCES**

1. Shepherd F, Winton T, Livingston R, Johnson D, Rigas J, Cormier Y, et al. The impact of adjuvant vinorelbine (VIN) and cisplatin (CIS) on quality of life (QoL): results from the National Cancer Institute of Canada Clinical Trials Group Trial BR.10 randomized trial of VIN/CIS versus observation in stage IB and II non-small cell [abstract]. *Ann Oncol* (Meeting Abstracts). 2004;15(Suppl 3):A627O.

2. Winton T, Livingston R, Johnson D, Rigas J, Johnston M, Butts C, et al. Vinorelbine plus cisplatin vs. observation in resected non-small-cell lung cancer. *New Engl J Med*. 2005;352(25):2589-97.

3. Scagliotti GV, De Marinis F, Rinaldi M, Crino L, Gridelli C, Ricci S, et al. Phase III randomized trial comparing three platinum-based doublets in advanced non-small cell lung cancer. *J Clin Oncol*. 2002;20(21):4285-91.

4. Douillard JY, Rosell R, De LM, Carpagnano F, Ramlau R, Gonzales-Larriba JL, et al. Adjuvant vinorelbine plus cisplatin versus observation in patients with completely resected stage IB-IIIA non-small-cell lung cancer (Adjuvant Navelbine International Trialist Association [ANITA]): a randomised controlled trial. *Lancet Oncol*. 2006;7(9):719-27.

5. Strauss GM, Herndon JE, Maddaus MA, Johnstone DW, Johnson EA, Watson DM, et al. Adjuvant chemotherapy in stage IB non-small cell lung cancer (NSCLC): Update of Cancer and Leukemia Group B (CALGB) protocol 9633. [abstract]. *J Clin Oncol*. 2006;A7007.

6. Strauss GM, Herndon JE, Maddaus MA, Johnstone DW, Johnson EA, Watson DM, et al. *Adjuvant chemotherapy in stage IB non-small cell lung cancer (NSCLC): Update of Cancer and Leukemia Group B (CALGB) protocol 9633* [slide presentation on the Internet]. ASCO.org Virtual Meeting. [cited 2006 Jul 28]. Available at: http://www.asco.org/portal/site/ASCO/menuitem.34d60f5624ba07fd506fe310ee37a01d/?vgnextoid=76f8201eb61a7010VgnVCM100000ed730ad1RCRD&vmview=abst_detail_view&confID=40&abstractID=31773.

**Related Guidelines**

Practice Guideline Report #7-1-1: *Postoperative Adjuvant Radiation Therapy in Stage II or IIIA Completely Resected Non-Small Cell Lung Cancer*.

*Funding*

The PEBC is supported by Cancer Care Ontario (CCO) and the Ontario Ministry of Health and Long-Term Care. All work produced by the PEBC is editorially independent from its funding agencies.

*Copyright*

This evidence-based series is copyrighted by Cancer Care Ontario; the series and the illustrations herein may not be reproduced without the express written permission of Cancer Care Ontario. Cancer Care Ontario reserves the right at any time, and at its sole discretion, to change or revoke this authorization.

*Disclaimer*

Care has been taken in the preparation of the information contained in this document. Nonetheless, any person seeking to apply or consult the evidence-based series is expected to use independent medical judgment in the context of individual clinical circumstances or seek out the supervision of a qualified clinician. Cancer Care Ontario makes no representation or guarantees of any kind whatsoever regarding their content or use or application and disclaims any responsibility for their application or use in any way.

*Contact Information*

For further information about this series, please contact

**Dr. William K. Evans**, Co-Chair, Lung Cancer Disease Site Group, McMaster University and Juravinski Cancer Centre, 699 Concession Street, Hamilton ON L8V 5C2; TEL (905) 387-9711 ext. 63001; FAX (905) 575-6323;

or

**Dr. Yee C. Ung**, Co-Chair, Lung Cancer Disease Site Group, Toronto-Sunnybrook Regional Cancer Centre, 2075 Bayview Ave, Toronto, ON M4N 3M5; TEL (416) 480-4951; FAX (416) 480-6002.

For information about the PEBC and the most current version of all reports,

please visit the CCO Web site at http://www.cancercare.on.ca/ or contact the PEBC office at: Phone: 905-525-9140, ext. 22055 Fax: 905-522-7681
