# Supplementary material for: A mixed methods approach to understand variation in lung cancer practice and the role of guidelines
Source: Implement Sci. 2014 Mar 22;9:36. doi: 10.1186/1748-5908-9-36 (PMC3998045; doi:10.1186/1748-5908-9-36)
Supplement: Additional file 2 — Practice Guideline 2: Unresected NSCLC Stage IIIA and IIIB Patients. [file 1748-5908-9-36-S2.docx]

**Additional file 2: Practice Guideline B: Unresected NSCLC Patients**

**Management of Unresected Stage Ill Non-Small Cell Lung Cancer (7-3)**

**Guideline Questions**

1. What is the role of different schedules or doses of radiotherapy as a treatment in patients with unresected stage Ill non-small cell lung cancer?
2. Does chemotherapy combined with radiotherapy improve survival compared with radiotherapy alone in patients with unresected stage III non-small cell lung cancer?
3. Which sequence of radiotherapy combined with chemotherapy is most effective in improving survival for patients with unresected stage III non-small cell lung cancer?
4. Which chemotherapy regimen(s), combined with radiotherapy, is most effective in improving survival for patients with unresected stage III non-small cell lung cancer?

**Target Population**

These recommendations apply to adult patients with unresected, clinical or pathological, stage III non-small cell lung cancer. Unresected disease is defined as a tumour that, for either technical or medical reasons, cannot be completely resected or removed.

**Recommendations**

► For patients with good performance status (Eastern Cooperative Oncology Group, 0-1) and minimal weight loss (usually defined as <5% in the preceding three months):

- Chemoradiation improves survival compared with radiotherapy alone and concurrent chemoradiation is recommended, with cisplatin-based chemotherapy and thoracic radiation of at least 60 Gy in 30 fractions given over a six-week period.
- Insufficient evidence exists to recommend a specific cisplatin-based regimen for use in a concurrent chemoradiation schedule. However, in the opinion of the Lung Cancer Disease Site Group, reasonable treatment options include cisplatin combined with one of etoposide, vinorelbine, or vinblastine.

► For symptomatic patients with poor performance status (Eastern Cooperative Oncology Group, >1) and significant weight loss (usually defined as >10% in the preceding three months):

- Radiotherapy for symptom palliation is recommended.
- Insufficient evidence exists to determine the optimal dose or timing of radiotherapy when the goal of therapy is symptom palliation. Reasonable treatment options include 20 Gy in five fractions and 17 Gy in two fractions given one week apart. Radiotherapy administered in a single fraction of 10 Gy is not recommended based on the decreased survival and quality of life observed when compared with multifractionated radiotherapy in one Canadian trial. However, in the opinion of the Lung Cancer Disease Site Group, single fractions of radiotherapy less than 10 Gy may be appropriate in some clinical circumstances.
- Palliative chemotherapy for patients with stage III disease is not reviewed in this guideline. For guidelines on palliative chemotherapy for locally advanced (stage IIIB) or metastatic (stage IV) disease, please visit the Cancer Care Ontario Web site.

► For patients with borderline performance status or moderate weight loss (5-10%):

- Concurrent or sequential chemoradiation is an option though the quality and quantity of evidence is not as compelling as that for patients with good performance status and minimal weight loss.

► Hyperfractionated radiation is not recommended outside the context of a clinical trial

**Qualifying Statements**

- Full dose vinorelbine (25-30 mg/m2 weekly) should not be used in combination with cisplatin and concurrent radiotherapy because of toxicity concerns. The two trials of concurrent cisplatin-vinorelbine and radiotherapy reviewed in this guideline used a vinorelbine dose of 12.5-15 mg/m2 generally administered weekly.
- Evidence for the use of induction chemotherapy before concurrent chemoradiation is currently limited; therefore, the Lung Cancer Disease Site Group believe that, where concurrent chemoradiation is used, the two treatment modalities should be *started* at the same time and as early as possible after diagnosis.
- Insufficient evidence exists to recommend for or against the use of chemotherapy as consolidation treatment after chemoradiation. However, based on the limited evidence available, if consolidation chemotherapy is used, two to three cycles could be administered.
- Increased toxicity, particularly esophagitis and hematologic events, is associated with the addition of chemotherapy to radiotherapy. The results of one randomized phase II trial comparing three different cisplatin-based doublets combined with concurrent radiotherapy suggest that these toxicities occur more frequently with gemcitabine-cisplatin compared with paclitaxel-cisplatin or vinorelbine-cisplatin.

**Key Evidence**

- Fifteen randomized trials examined various policies or dose and schedule combinations for radiotherapy administration. Among the fully published trials, no statistically significant survival differences were detected for immediate versus delayed administration of radiotherapy (one trial) or different doses of hyperfractionated radiotherapy (one trial). Of the 11 fully published trials that compared varying doses and schedules of standard fractionated radiotherapy, three detected a statistically significant survival advantage with higher multifractionated radiation doses (20 Gy in five fractions versus 10 Gy in one fraction, p=0.03; 39 Gy in 13 fractions versus 17 Gy in two fractions, p=0.03; 30 Gy in 10 fractions versus 16 Gy in two fractions, p=0.03). One trial detected a survival advantage for 16 Gy in two fractions compared with 20 Gy in five fractions (p=0.016); however, the reliability of this result is limited by the size of the trial (n=100) and the fact that survival was a secondary outcome.
- Six meta-analyses compared chemoradiation with radiation alone; three focused exclusively on trials that administered concurrent chemoradiation. There was considerable overlap in the studies included in each meta-analysis, and the results were consistent. The largest meta-analysis involved 22 studies and individual patient data from more than 3,000 patients. That meta-analysis detected a statistically significant overall survival benefit for the use of chemoradiation with a pooled hazard ratio of 0.90 (p=0.006) or a 10% relative reduction in the risk of death, which translated into an absolute benefit of 3% at two years and 2% at five years. The survival benefit associated with chemoradiation was maintained in a subgroup analysis of 11 trials involving cisplatin-based chemoradiation (pooled hazard ratio, 0.87; 95% confidence interval, 0.79 to 0.96), increasing survival from 15% to 19% at two years (absolute benefit, 4%) and from 5% to 7% at five years (absolute benefit, 2%). Chemotherapy combinations not including cisplatin did not demonstrate a statistically significant survival benefit.
- Two of seven trials compared radiotherapy alone to radiotherapy combined with low-dose platinum-based chemotherapy used as a radiosensitizer and detected a statistically significant survival advantage for chemoradiation (estimated three-year survival: 16% versus 2%, p=0.009; 10% versus 2%, p=0.00001). Both trials used concurrent daily cisplatin as the radiosensitizer; however, one trial also used split-course radiotherapy, which is generally considered less effective than continuous radiotherapy because of the theoretical possibility of tumour repopulation during the rest period.
- Of seven trials that compared conventional radiotherapy alone with chemoradiation, longer survival was generally associated with the combined treatment, although the difference was statistically significant in only one small trial (n=51). Two of four small trials (32-68 patients per treatment arm) that added platinum-based chemotherapy to hyperfractionated or accelerated radiotherapy detected a statistically significant survival advantage for the combination treatment compared with radiotherapy alone (median: 18 versus 8 months, p=0.0027; 22 versus 14 months, p=0.021).
- One meta-analysis also detected a survival benefit for concurrent compared with sequential chemoradiation at two years (n=711; relative risk, 0.86; 95% confidence interval, 0.78-0.95; p=0.003).
- Three randomized trials, one reported in abstract form, comparing sequential to concurrent chemoradiotherapy detected a statistically significant survival advantage for concurrent treatment (median: 16.5 versus 13.3 months, p=0.04; 16.6 versus 12.9 months, p=0.023; 17.0 versus 14.6 months, p=0.046). Three additional trials compared sequential or concurrent chemoradiation with concurrent chemoradiation followed by consolidated chemotherapy or preceded by induction chemotherapy. In the two trials providing a statistical comparison of survival, no significant differences were detected between treatment schedules.
- Of six fully published trials that compared different chemotherapy regimens or schedules within a combined modality treatment approach, three involved older or non-standard chemotherapy regimens combined with split-course radiotherapy, one involved hyperfractionated chemoradiation with or without weekend chemotherapy and detected no statistically significant survival differences between groups, one compared concurrent with sequential chemoradiation involving two different chemotherapy regimens, and one involved three newer platinum-based chemoradiation combinations, but that trial was not designed to compare survival across treatment groups.
- Clinical experience suggests that the toxicity resulting from chemotherapy and/or radiotherapy in the treatment of unresected stage III NSCLC is largely confined to neutropenic-related infection, weight loss, and vomiting. Weight loss and serious infections requiring hospitalization are more prevalent with chemoradiation (sequential or concurrent) compared to radiation alone. Patients receiving concurrent combined chemoradiation or radiation alone are at risk for radiation pneumonitis and esophagitis, and meta-analyses indicate that severe acute esophagitis is more frequently associated with concurrent chemoradiation compared with radiation alone. Toxicities commonly reported in clinical trials include hematologic toxicity and nausea and vomiting, both associated with combined chemoradiation. Symptom control and quality of life were mainly assessed in trials comparing different radiotherapy schedules and doses, with few reporting a statistically significant difference between schedules.

**Related Guidelines**

Practice Guideline Report #7-12: Altered Fractionation of Radical Radiation Therapy in the Management of Unresectable Non-Small Cell Lung Cancer.

*Funding*

The PEBC is supported by Cancer Care Ontario (CCO) and the Ontario Ministry of Health and Long-Term Care. All work produced by the PEBC is editorially independent from its funding agencies.

*Copyright*

This evidence-based series is copyrighted by Cancer Care Ontario; the series and the illustrations herein may not be reproduced without the express written permission of Cancer Care Ontario. Cancer Care Ontario reserves the right at any time, and at its sole discretion, to change or revoke this authorization.

*Disclaimer*

Care has been taken in the preparation of the information contained in this document. Nonetheless, any person seeking to apply or consult the evidence-based series is expected to use independent medical judgment in the context of individual clinical circumstances or seek out the supervision of a qualified clinician. Cancer Care Ontario makes no representation or guarantees of any kind whatsoever regarding their content or use or application and disclaims any responsibility for their application or use in any way.

*Contact Information*

For further information about this series, please contact

**Dr. William K. Evans**, Co-Chair, Lung Cancer Disease Site Group, McMaster University and Juravinski Cancer Centre, 699 Concession Street, Hamilton ON L8V 5C2; TEL (905) 387-9711 ext. 63001; FAX (905) 575-6323;

or

**Dr. Yee C. Ung**, Co-Chair, Lung Cancer Disease Site Group, Toronto-Sunnybrook Regional Cancer Centre, 2075 Bayview Ave, Toronto, ON M4N 3M5;

TEL (416) 480-4951; FAX (416) 480-6002.

For information about the PEBC and the most current version of all reports,

please visit the CCO website at http://www.cancercare.on.ca/

or contact the PEBC office at:

Phone: 905-527-4322 ext. 42822 Fax: 905-526-6775 E-mail: ccopgi@mcmaster.ca
