# Supplementary material for: A mixed methods approach to understand variation in lung cancer practice and the role of guidelines
Source: Implement Sci. 2014 Mar 22;9:36. doi: 10.1186/1748-5908-9-36 (PMC3998045; doi:10.1186/1748-5908-9-36)
Supplement: Additional file 3 — Interview Question Guide. [file 1748-5908-9-36-S3.docx]

**Additional file 3: Interview Question Guide**

**Interview Question Guide – For Physicians**

**Treatment Decision-Making Process**

1) Could you tell me a little bit about how you generally approach treatment decision-making with your non-small cell lung cancer patient (specifically in stage II and stage IIIA resected; and stage IIIA and stage IIIB non-resected)?

a. Probe: How did you approach a recent case? (Give a few examples)

b. Probe: Have other cases gone differently?

c. Probe: What does your treatment decision making process usually involve? (i.e.

examples of other patient treatments and their relevance of the patient in front of

you, patient preferences, CCO guidelines, standard treatment, personal, social,

clinical situation of the patient?)

d. Probe: What do you typically recommend to your stage II and stage IIIA

resected non-small cell lung cancer patients? And what do you typically

recommend to your stage IIIA and stage IIIB non-resected non-small cell lung

cancer patients?

e. Probe: Are the discussions with these patients challenging or uncomfortable?

**Regarding Data and Guidelines**

1) Can you please explain to me how familiar you are with the clinical evidence, and

with the primary clinical studies regarding the treatment of non-small cell lung

cancer patients?

2) How are you made aware of these PEBC/CCO clinical guidelines?

a. Probe: How are you alerted of this information?

b. Probe: How do you access this information? (i.e. larger cancer centres may

have CCO representatives, but what about smaller cancer centres? What are

the mechanisms in communicating this information: reps, leaders, disease

group?)

3) What are your thoughts on the PEBC/CCO clinical guidelines and from the evidence

they are derived?

a. Probe: Do you find the evidence compelling?

b. Probe: What is your opinion of the research studies on which these treatment

recommendations are based?

4) How generalizable, in your opinion are the findings of these research studies?

i. Probe: Share an example of one or more cases when treating patients with

non-small cell lung cancer (stage II and stage IIIA resected/ and stage IIIA

and stage IIIB non-resected) where you had made treatment decisions

consistent with the PEBC/CCO guidelines.

ii. Probe: Share an example of one or more cases when treatment patients

with non-small cell lung cancer (stage II and stage IIIA resected/ and stage

IIIA and stage IIIB non-resected) where you had made treatment decisions

but deviated from the PEBC/CCO guidelines.

5) What are the consequences of these guidelines being transferred into actual care?

For the patient? For the physician? For other aspects of care?

6) Based on the data and the recommendations, do you perceive a practice gap or

quality of care issue?

**Regarding Adherence to Guidelines**

1) Do you think that recommendations should be followed? Are they being followed enough, too much, too little?

2) *If the respondent agrees to the above question, and agrees that recommendations should be followed more*:

What kinds of things might make these recommendations easier to follow?

o Probe: What tools and strategies would be acceptable to you?

o Probe: What is feasible in your practice setting?

**Regarding Current Practice**

1) *Surgeons or community medical oncologists*: Do you refer patients to the Cancer Centre for a consultation? Why or why not?

a. Probe: How does the Cancer Centre respond to your referral? (I.e. do they

respond in a timely fashion?)

b. Probe: Do you see a pattern of patient preference that does not align with

PEBC/CCO treatment recommendations?

2) Explain any issues around access to treatments for non-small cell lung cancer? (i.e.

affordability – so costs of travel for the patient to get to treatment, physical

accessibility - the patients are unable to get to treatment centres, acceptability of

services, adequacy of supply)

**Regarding Support Practice**

1) How do you provide information or education to your patients?

a. Probe: What kinds of information resources do you use most to support your

treatment discussions with your patients?

2) How do your patients respond to your use of educational information resources?

3) Does your clinic support the care and treatment of non-small cell lung cancer

patients?

**Ending Questions**

1. The purpose of this interview was to hear your thoughts about current clinical practice guidelines for non-small cell lung cancer patients, hear more about your current practice patterns, and better understand how physicians are making treatment decisions with their non-small cell lung cancer patients. Is there anything more you would like to add? What other questions should we be asking about non-small cell lung cancer treatment and the Cancer Care Ontario guidelines?

**Interview Question Guide – For Administrators**

**Regarding PEBC, Data and Guidelines**

1. How familiar are you with the Program in Evidence-based Care in general?

2. Are you familiar with their guideline methodology process?

3. Are you familiar with the principles of evidence-informed guideline development?

4. Have you read a PEBC guideline before?

5. Are you aware of when new documents are released?

6. Is there a mechanism by which your region is made aware of a new or updated PEBC guideline

a. For example, is someone responsible for disseminating them to you, other

administrative leaders, and the clinicians in your region?

b. Are there strategies you would like to see developed so that you are made

aware of guidelines and the recommendations?

7. Do you use the guidelines to help justify or inform the development or implementation

of quality initiatives within your region (OR IF IT’S A PROVINCIAL LEADER – within

your provincial program)?

8. Do the clinical leaders in your region use PEBC guidelines to improve quality of care?

*DEPENDING ON WHAT THEY ANSWER ABOVE*

9. Are you aware of the guidelines related to the treatment of non-small cell lung cancer

patients?

10. If yes,

a. Are you aware of the recommendations?

b. Do you have an opinion about the quality of these guidelines or the quality

of the evidence-base underpinning the recommendations?

**Performance Data**

My project is in response to recent data from the Cancer System Quality Index (CSQI) of practice patterns regarding treatment recommendations for Non-small cell lung cancer Patients [7-1-2 & 7-3]. These recommendations are:

- Patients with stage II and stage IIIA resected non-small cell lung cancer should receive cisplatin-based chemotherapy.
- Patients with stage IIIA and IIIB non-resected non-small cell lung cancer should receive chemo-radiotherapy with a cisplatin-based agent.

*SHOW THEM THE GRAPH or DESCRIBE IT*

11. So my question is: from an administrator perspective, what is your response to the above presented information?

a. Probe: Do you perceive a practice gap or quality of care issue across the

province?

b. Are you concerned with these patterns?

c. Are you concerned about the performance for your region?

d. Does this data suggestion action is required? Would this be action by

you?

e. Do you see a role for the PEBC guideline to help address this variation in

practice?

f. If yes,

i. How should that role be operationalize to optimize the value of a

PEBC guideline

ii. Probe: Whom do we need to communicate to? The individual

physicians? The multi-disciplinary groups as a whole?

Multidisciplinary cancer conferences? Who should communicate?

You, the clinical lead, the PEBC directly?

**Ending Questions**

12. The purpose of this interview was to hear your thoughts about current clinical

practice guidelines for non-small cell lung cancer patients, hear more about clinical practice patterns, and better understand your administrative perspective on the practice variation we are seeing for stage II and stage III non-small cell lung cancer patients across regions in Ontario. Is there anything more you would like to add? What other questions should we be asking about these recommendations, the treatment decision-making for non-small cell lung cancer patients, and current practice patterns to administrators, or physicians treating these specific patients?
